# Incomplete proline catabolism drives premature sperm aging

**DOI:** 10.1111/acel.13308

**Published:** 2021-01-21

**Authors:** Chia‐An Yen, Sean P. Curran

**Affiliations:** ^1^ Leonard Davis School of Gerontology University of Southern California Los Angeles CA USA; ^2^ Department of Molecular and Computation Biology Dornsife College of Letters, Arts, and Sciences University of Southern California Los Angeles CA USA

**Keywords:** aging, *alh‐6*/ALDH4A1, antioxidants, *C. elegans*, germ cells, male‐specific, mitochondria, N‐acetylcysteine, P5C dehydrogenase, proline catabolism, reactive oxygen species, reproduction, senescence, spermatogenesis, vitamin C

## Abstract

Infertility is an increasingly common health issue, with rising prevalence in advanced parental age. Environmental stress has established negative effects on reproductive health, however, the impact of altering cellular metabolism and its endogenous reactive oxygen species (ROS) on fertility remains unclear. Here, we demonstrate the loss of proline dehydrogenase, the first committed step in proline catabolism, is relatively benign. In contrast, disruption of *alh*‐*6*, which facilitates the second step of proline catabolism by converting 1‐pyrroline‐5‐carboxylate (P5C) to glutamate, results in premature reproductive senescence, specifically in males. The premature reproductive senescence in *alh*‐*6* mutant males is caused by aberrant ROS homeostasis, which can be countered by genetically limiting the first committed step of proline catabolism that functions upstream of ALH‐6 or by pharmacological treatment with antioxidants. Taken together, our work uncovers proline metabolism as a critical component of normal sperm function that can alter the rate of aging in the male reproductive system.

## Graphical abstract

The loss of *alh‐6*, a nuclear encoded mitochondrial enzyme that catalyzes the second step of proline catabolism by converting 1‐pyrroline‐5‐carboxylate (P5C) to glutamate, disrupts endogenous redox imbalance and causes premature reproductive aging in *C. elegans* males. Mutation in *prdh‐1*, the upstream enzyme in the proline catabolic pathway which breaks down proline to P5C, suppresses the sperm defects in *alh‐6* mutant males by preventing the accumulation of the toxic intermediate P5C. Similarly, dietary antioxidant treatment rescues the premature aging defects in alh‐6 mutant sperm, implicating the role of proline catabolism in maintaining endogenous ROS homeostasis and male reproductive senescence.

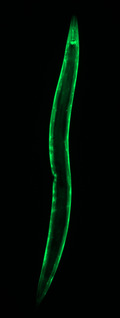

## 1 INTRODUCTION

Infertility is defined as the inability for a couple to conceive within a year of unprotected sex. There is an estimated number of 12–13% of couples in the United States that struggle with infertility (Chandra et al., 2014). While emphasis on female factors in fertility is important and their roles have been extensively studied, male factors play an equally important role in determining the outcome of a successful fertilization. Male fertility is often measured as a function of sperm quality and quantity, since these factors are correlated with time to pregnancy and pregnancy success (Buck Louis et al., 2014). As an increasing number of couples wait to have children, age becomes a risk factor for infertility problems; the increase in paternal age, much like maternal age, is also associated with adverse gamete health, negative pregnancy outcome, and increased risk of birth defects (Sharma et al., 2015).

In sexually reproducing species, sperm competition plays an important role in reproductive fitness. In species where females mate with multiple males, a male can improve his reproductive success if his sperm outcompetes sperm from other males in fertilizing the oocyte of a female. This competitive edge can be achieved by males through producing large quantities of sperm or generating higher‐quality sperm (Ramm et al., 2014). In *Caenorhabditis elegans*, adolescent hermaphrodites produce sperm before switching to oogenesis in adulthood. When mated with males, hermaphrodite‐derived sperm are disadvantaged and outcompeted by male sperm which are greater in size and speed (LaMunyon & Ward, 1998). In addition to hermaphrodite‐male sperm competition, male‐male sperm competition can also occur when a hermaphrodite is mated with multiple partners. As such, sperm quality is a competitive parameter of overall fitness in sexually reproducing species.

Mitochondria are essential for their role in fueling cellular functions. Notably, multiple studies in humans and mice have implicated different aspects of mitochondrial function in sperm quality including mitochondria ultrastructure, mitochondrial genome and copy number (LaMunyon & Ward, 1998), mitochondrial protein levels, as well as enzyme activity of the electron transport chain (ETC) complexes (Amaral et al., 2013). While all these studies imply that mitochondrial integrity and activity are critical for proper sperm function, the mechanisms behind this relationship remain unclear.

While cells need mitochondria to generate energy, this process generates ROS as a natural byproduct (Murphy, 2009). Low levels of ROS are essential and play an important role in cell signaling, hypoxia adaptation, aging, autophagy, immunity, and cell differentiation (Sena & Chandel, 2012), while high levels of ROS can be detrimental to cellular function and can lead to cell death. In mammals, multiple aspects of sperm function and successful fertilization including capacitation, hyperactivation, acrosome reaction, and sperm‐oocyte fusion require low levels of ROS (Agarwal et al., 2014). Interestingly, many studies have found elevated ROS in sperm to be associated with increased lipid peroxidation, increased DNA damage, and reduced sperm motility and viability; although the source of ROS and the mechanism behind ROS‐induced sperm defects are unknown (Agarwal et al., 2014). Recent studies show that mitochondria‐generated ROS through inhibition of the ETC results in spermatozoa with reduced motility and increased lipid peroxidation in vitro (Aitken et al., 2012; Koppers et al., 2008). Since the level of ROS in semen also increases with age (Cocuzza et al., 2008), understanding ROS‐mediated sperm defects may provide insight into male reproductive senescence.

Proline plays a critical role in cellular metabolism and functions as a central amino acid in cellular bioenergetics and redox control (Phang, 1985), but has recently become recognized as a mediator of aging and age‐related conditions (Donald et al., 2001; Pang & Curran, 2014; Pang et al., 2014; Rivera & Maxwell, 2005; Yen et al., 2020). Catabolism of proline to glutamate is a two‐step metabolic process where proline is first converted to 1‐pyrroline‐5‐carboxylate (P5C) by proline dehydrogenase (PRDH‐1) and subsequently to glutamate by P5C dehydrogenase (ALH‐6). Mutation in *alh*‐*6* results in the accumulation of P5C, which generates ROS and leads to loss of cellular integrity (Nomura & Takagi, 2004; Pang & Curran, 2014; Pang et al., 2014). Our previous findings demonstrated that *alh*‐*6* mutation leads to the depletion of flavin adenine dinucleotide (FAD) reserves and drives changes in mitochondrial dynamics, leading to sperm dysfunction (Yen et al., 2020). Here, we investigate the role of the enzyme upstream in the proline catabolism pathway, PRDH‐1, in regulating sperm health as a function of age.

## 2 RESULTS

### 2.1 Mutation in proline dehydrogenase suppresses *alh*‐*6* mutant phenotypes

Our earlier studies revealed that mutation in *alh*‐*6* activates the SKN‐1 activity reporter *gst*‐*4p*::*gfp* and demonstrated that the ALH‐6 pathway regulates lifespan and healthspan in *C. elegans*, in part, through its ability to catabolize P5C to maintain cellular redox homeostasis (Pang & Curran, 2014). Recently, we showed that the *alh*‐*6* mutation also leads to changes in FAD abundance and mitochondrial homeostasis resulting in impaired male reproductive fitness in *C. elegans* (Yen et al., 2020). To further understand how disruption of proline catabolism can influence these health outcomes, we sought to identify genetic regulators of the stress response stemming from the mutation in *alh*‐*6*.

**FIGURE 1:**
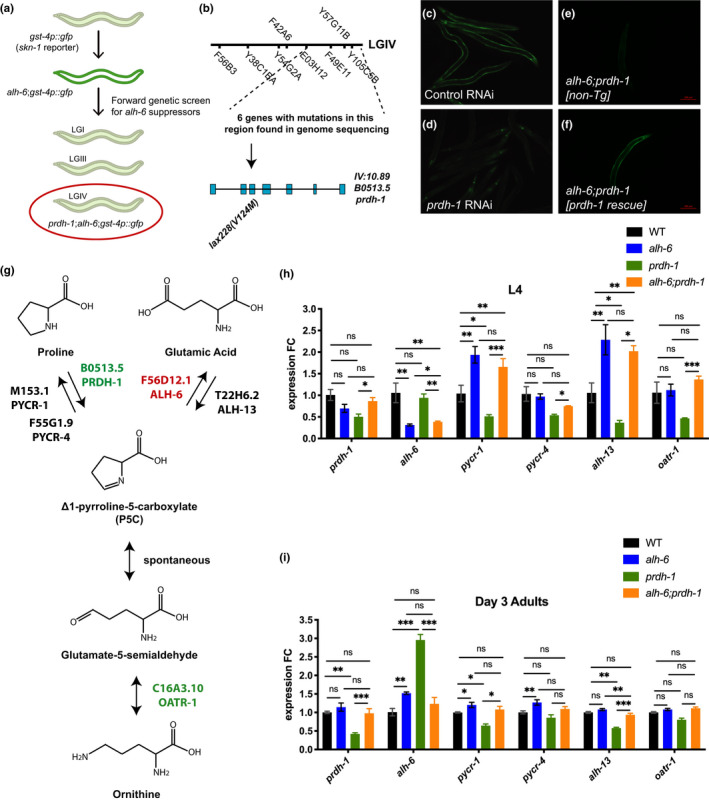
*prdh*‐*1* mutation suppresses activation of SKN‐1 and proline metabolism deregulation in older *alh*‐*6* animals. (a) Cartoon depiction of EMS screen for suppressors of SKN‐1 reporter activation in *alh*‐*6* mutants. (b) SNP mapping identifies linked loci of *prdh*‐*1(lax228)* marked by dashed lines. Mutation locus of *prdh*‐*1(lax228)* in the gene is marked by arrow. (c, d) RNAi knockdown of *prdh*‐*1* suppresses the activation of SKN‐1 in *alh*‐*6*;*gst*‐*4p*::*gfp* animals. (c) Day 3 adult *alh*‐*6*;*gst*‐*4p*::*gfp* fed L4440 (control RNAi). (d) Day 3 adult *alh*‐*6*;*gst*‐*4p*::*gfp* fed *prdh*‐*1* RNAi. (e, f) *prdh*‐*1* rescue reverts suppression of SKN‐1 activation in *alh*‐*6(lax105)*;*prdh*‐*1(lax228)*;*gst*‐*4p*::*gfp* animals. (e) Day 3 *alh*‐*6(lax105)*; *prdh*‐*1(lax228)*;*gst*‐*4p*::*gfp* adults. (f) Day 3 *alh*‐*6(lax105)*;*prdh*‐*1(lax228)*;*gst*‐*4p*::*gfp* adults with *prdh*‐*1* rescue construct. (g) Schematic of biosynthetic and catabolic pathways of proline in *C. elegans*. (h,i) RT‐PCR analysis of gene expression changes in the proline metabolism pathway in L4 and Day 3 adult hermaphrodites. (h) *alh*‐*6* single and *alh*‐*6*;*prdh*‐*1* double mutant animals show similar increased expression in proline biosynthesis genes at L4 stage. (i) Day 3 *alh*‐*6* mutant animal upregulate genes such as *alh*‐*6*, *pycr*‐*1*, and *pycr*‐*4* in concerted effort to detoxify P5C. Notably, older *alh*‐*6*;*prdh*‐*1* double mutants show WT level of expression in proline metabolism genes. Statistical comparisons of RT‐PCR results in worms were done using ANOVA between all groups. *, *p* < 0.05; **, *p* < 0.01; ***, *p* < 0.001; ****, *p* < 0.0001. All studies performed in minimum of biological three triplicates; refer to Table S1 for *n* for each comparison

We performed an ethyl methanesulfonate (EMS) mutagenesis screen to identify suppressors of the age‐dependent induction of the *gst*‐*4p*::*gfp* reporter that defines *alh*‐*6* mutant animals (Figure 1a) (Pang & Curran, 2014; Yen et al., 2020). One complementation group, defined by the suppressor allele *lax228*, was mapped to the right arm of chromosome IV between the DraI cut sites defined by the single nucleotide polymorphisms (SNP) E03H12 and Y105C5B (Figure 1b). We performed whole‐genome sequencing to identify non‐synonymous mutations in the exons of protein‐coding genes in this region and compiled a list of candidate genes (Figure S1a) (Blankenberg et al., 2010). We used RNA interference (RNAi) to knockdown each of these genes in the *alh*‐*6(lax105)*;*gst*‐*4p*::*gfp* strain to identify the gene that phenocopies the effects of the *lax228* suppressor allele. RNAi of *B0513*.*5*, hereafter referred to as *prdh*‐*1* as it encodes a putative proline dehydrogenase enzyme homologous to mammalian *Prodh*, was the only RNAi target in this region that phenocopied the *lax228* mutant (Figure 1c,d, Figure S1b). Furthermore, transgenic restoration of wild‐type (WT) *prdh*‐*1* reverts the suppression of SKN‐1 activation observed in *alh*‐*6(lax105)*;*prdh*‐*1(lax228)*;*gst*‐*4p*::*gfp* to levels comparable to *alh*‐*6(lax105)* single mutants (Figure 1e,f). Proline dehydrogenase (*prdh*‐*1)* catalyzes the first enzymatic step of proline catabolism (Figure 1g), converting proline to P5C (Adams & Frank, 1980). Thus, mutation of *prdh*‐*1* prevents the accumulation of the toxic intermediate P5C, which leads to activation of SKN‐1 in *alh*‐*6* mutants (Figure S1b). Since *alh*‐*6* mutation causes sperm defects, we checked the activity of SKN‐1 in males harboring the *gst*‐*4p*::*gfp* reporter. Interestingly, SKN‐1 is activated in *alh*‐*6(lax105)*;*gst*‐*4p*::*gfp* males as early as day 1 of adulthood, which is when sperm defects are manifested (Figures S1c). *prdh*‐*1* mutation suppresses the activation of SKN‐1 by *alh*‐*6* mutation in both hermaphrodites and males by day 3 of adulthood (Figures S1b,d). Next, we followed the suppression of activated SKN‐1 while backcrossing the *alh*‐*6(lax105)*;*prdh*‐*1(lax228)*;*gst*‐*4p*::*gfp* strain to *alh*‐*6(lax105)* mutants six times. We then sequence verified *lax228* allele to be a G to A transition mutation in exon 3 of *prdh*‐*1* that results in a valine to methionine (V124M) substitution (Figure 1b). Finally, we performed two additional backcrosses to remove the *gst*‐*4p*::*gfp* reporter to generate the *alh*‐*6(lax105)*;*prdh*‐*1(lax228)* strain SPC494; referred hereafter as *alh*‐*6*;*prdh*‐*1*.

### 2.2 Temporal regulation of gene expression in the mitochondrial proline catabolism pathway

Proline level is regulated transcriptionally through concerted activities of biosynthetic and catabolic enzymes in the pathway (Rizzi et al., 2015). In addition, stress from environment or changes in metabolism can alter levels of proline through transcriptional activation or repression of these enzymes. (Mizoi & Yamaguchi‐Shinozaki, 2013; Verbruggen & Hermans, 2008). We examined the expression of the proline catabolism pathway genes via RT‐PCR (Figure 1h) and saw a significant change in the expression of enzymes that favor proline biosynthesis in *alh*‐*6* mutant larval stage 4 (L4) hermaphrodites, confirming our previous RNA‐Seq analysis (Yen et al., 2020). The *lax105* allele, containing a G to A missense mutation in exon 7 of *alh*‐*6* resulting in a glycine to glutamic acid (G521E) substitution, is likely hypomorphic based on previously observed phenotypes, including RNAi (Pang & Curran, 2014; Yen et al., 2020). Intriguingly, at the L4 stage, when spermatogenesis occurs, *alh*‐*6* mutant animals display a decrease in *alh*‐*6* expression, which would further contribute to P5C accumulation (Figure 1h). However, the expression of pyrroline‐5‐carboxylate reductase (*pycr*‐*1*/*PYCR*), which converts P5C back to proline, was increased, which could serve to counteract the accumulation of P5C. Surprisingly, the expression of pyrroline‐5‐carboxylate synthase (*alh*‐*13*/*P5CS)* was also increased in *alh*‐*6*; however, P5CS has two enzymatic functions: glutamate kinase (GK) and γ‐glutamyl phosphate reductase (GPR) activities, which impact additional nodes of cellular metabolism (Seddon et al., 1989; Wellner et al., 1974). We also found that the expression of proline biosynthesis genes *pycr*‐*1* and *alh*‐*13* are downregulated in *prdh*‐*1* mutants compared to WT animals, which may be a response to an increase in cellular proline level. Intriguingly, *alh*‐*6*;*prdh*‐*1* mutants at the L4 stage show upregulation of *pycr*‐*1* and *alh*‐*13* genes, which highlights a similar early transcriptional response as *alh*‐*6* single mutants. The transcriptional changes in proline metabolism genes in *prdh*‐*1* single mutants at the L4 stage are in the opposite direction from those of *alh*‐*6*;*prdh*‐*1* double mutants; proline biosynthesis genes *pycr*‐*1* and *alh*‐*13* are downregulated in *prdh*‐*1* single mutant, both of which are upregulated in *alh*‐*6*;*prdh*‐*1* double mutant. These findings suggest that mutation in *alh*‐*6* and the reduced ability to break down P5C, direct the early transcriptional response to increase cellular proline level by upregulating genes responsible for proline biosynthesis.

Next, we measured proline metabolism genes at day 3 of adulthood, when activation of the SKN‐1 reporter is observed in *alh*‐*6* animals, but suppressed in the *alh*‐*6*;*prdh*‐*1* double mutants (Figure 1i). Unlike in developing animals, where transcriptional changes were favored toward proline biosynthesis, day 3 adult *alh*‐*6* mutants show transcriptional changes in proline metabolism pathway that are in converted effort to remove toxic P5C. Noticeably, the expression of P5C dehydrogenase *alh*‐*6* and both P5C reductase genes, *pycr*‐*1*/*M153*.*1* and *pycr*‐*4*/*F55G1*.*9*, are all upregulated in *alh*‐*6* mutants. Notably, *alh*‐*6*;*prdh*‐*1* double mutant shows no transcriptional differences from WT. In *prdh*‐*1* single mutants, expression of proline biosynthetic genes *pycr*‐*1* and *alh*‐*13* remain low, while the expression of *alh*‐*6* is increased, probably due to the increased proline level in the animal and reduced ability for *prdh*‐*1* to break down proline. When comparing L4 animals to Day 3 adults for each genotype, the expression of all proline metabolism genes are downregulated with age (Figure 1h,i, Figure S1e–j). Based on these transcriptional responses observed at the L4 and day 3 adulthood stages in *alh*‐*6* and *alh*‐*6*;*prdh*‐*1* mutants, we next sought to understand the physiological consequence of having mutations in both *alh*‐*6* and *prdh*‐*1*.

### 2.3 Mutation of the first enzymatic step of proline catabolism is benign

**FIGURE 2:**
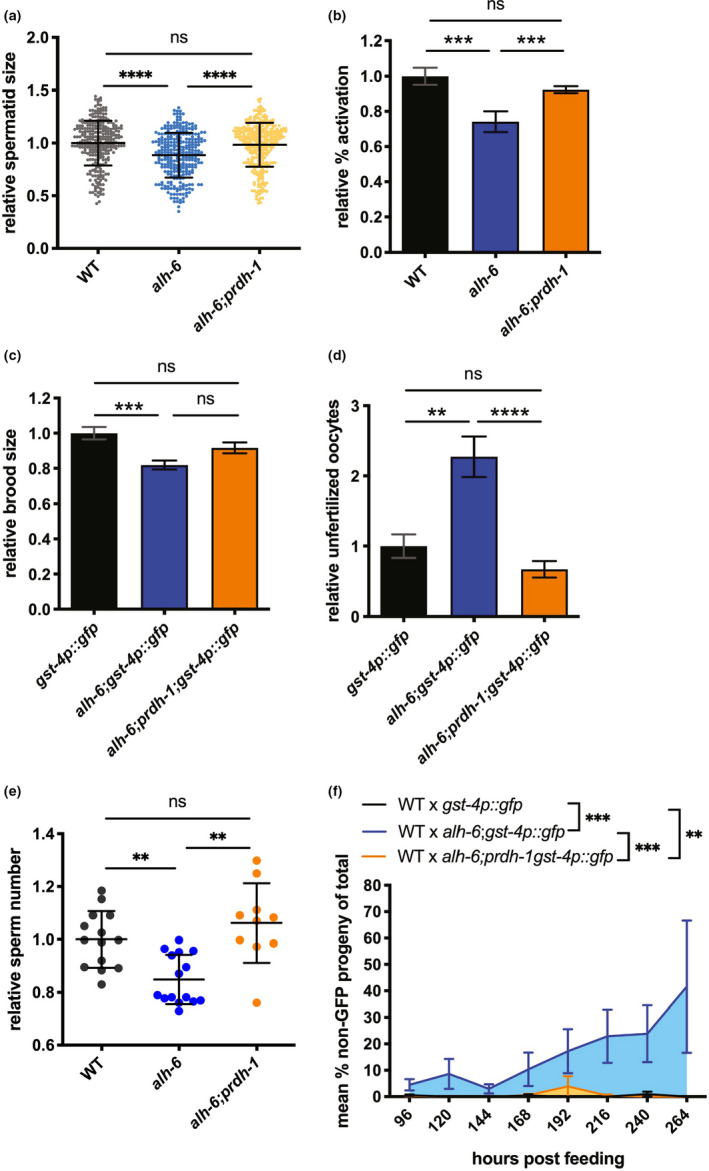
*prdh*‐*1* activity is required for sperm‐specific fertility defects in *alh*‐*6* mutants. (a–f) *prdh*‐*1* mutation rescues reduced male sperm size (a), impaired male sperm activation (b), reduced brood size in hermaphrodite (c), increased number of unfertilized oocytes in hermaphrodite (d), reduced sperm number in hermaphrodite (e), and male sperm competition (f) in *alh*‐*6* animals. Statistical comparisons of sperm size, progeny, unfertilized oocytes, and sperm number were done by ANOVA. Comparisons of sperm activation and competition assays were done using Fisher's exact test with adjusted p‐value cutoffs. *, *p* < 0.05; **, *p* < 0.01; ***, *p* < 0.001; ****, *p* < 0.0001. All studies performed in minimum of three biological triplicates; refer to Table S1 for n for each comparison

Because of the impact that *alh*‐6 mutation has on the expression of other proline catabolism pathway genes during development, we examined how mutation of *prdh*‐*1*, the first committed step of proline catabolism, would impact spermatid development and function. Our previous studies identified a role for *alh*‐*6* in sperm quality, and as such, we examined the *alh*‐*6*;*prdh*‐*1* double mutant animals in a panel of reproduction and sperm quality assays (Yen et al., 2020). The reduction in spermatid size (Figure 2a) and impairment of spermatid activation (Figure 2b) in *alh*‐*6* mutant males are both restored to WT levels when combined with a mutation in *prdh*‐*1*. Additionally, in the context of *alh*‐*6* single mutant hermaphrodite, loss of *prdh*‐*1* results in a trend toward increased fertility (Figure 2c), suppresses the number of unfertilized oocytes (Figure 2d), and increases sperm count (Figure 2e). Finally, the reduced ability of *alh*‐*6* male sperm to compete against wild‐type hermaphrodite sperm was abrogated in the *alh*‐*6*;*prdh*‐*1* double mutant (Figure 2f), but neither mutation results in an observable difference in the structure of the male copulatory organ (Figure S1k). Taken together, these results reveal that disruption of the first committed step of mitochondrial proline catabolism pathway is benign for animal reproductive fitness, but suggests instead that P5C accumulation is instrumental in driving sperm dysfunction in *alh*‐*6* animals.

### 2.4 Endogenous ROS drives *alh*‐*6* sperm defects

**FIGURE 3:**
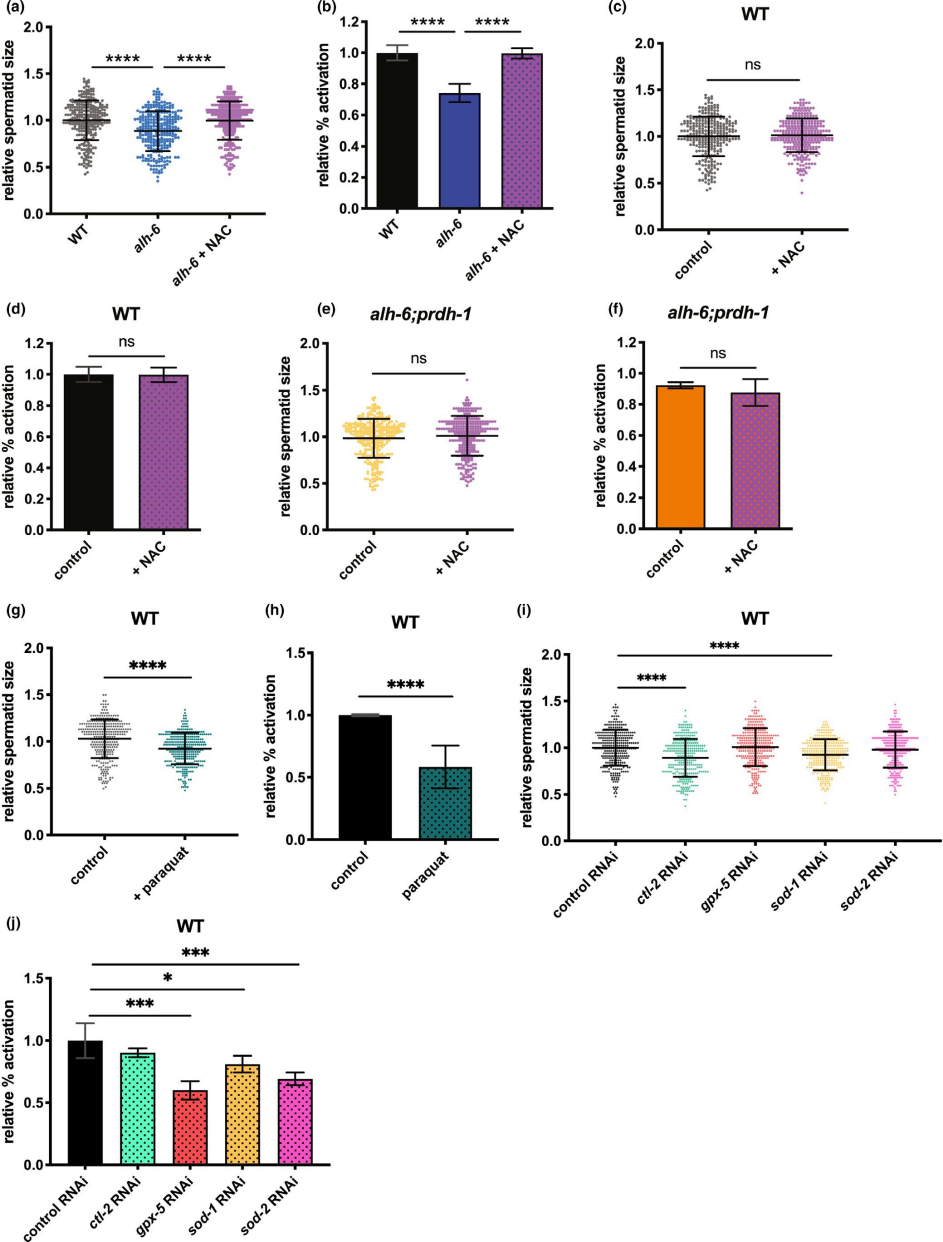
Endogenous ROS drives sperm defects in *alh*‐*6* mutant males. (a, b) Antioxidant NAC supplement restores sperm size (a) and sperm activation (b) in *alh*‐*6* mutants. (c, d) NAC supplementation does not affect sperm size (c) or sperm activation (d) in WT males. (e, f) NAC supplementation does not affect sperm size (e) or sperm activation (f) in *alh*‐*6*;*prdh*‐*1* males. (g, h) Paraquat treatment reduces size (g) and impairs activation (h) in WT males. (i‐j) RNAi of antioxidant enzymes in affecting sperm size (i) and activation (j) of WT males. Statistical comparisons of sperm size between WT and *alh*‐*6* were done using unpaired t‐test. Statistical comparisons of sperm size between a strain on diet supplemented with NAC versus vehicle control‐treated diet were also done with unpaired *t*‐test. Comparisons of sperm activation between WT and *alh*‐*6* or a strain on control diet versus supplemented diet were done using Fisher's exact test. Sperm size assays of WT on RNAi conditions were compared using ANOVA. Sperm activation assays of WT on RNAi conditions were compares using Fisher's exact test with adjusted p‐value cutoffs. *, *p* < 0.05; **, *p* < 0.01; ***, *p* < 0.001; ****, *p* < 0.0001. All studies performed in minimum of three biological replicates; refer to Table S1 for n for each comparison

Several studies have examined the impact of exogenous exposure to ROS‐inducing electrophiles on sperm function (de Lamirande & Gagnon, 1992; Oliveira et al., 2009), but the impact of endogenous ROS on sperm function remains poorly defined. Proline catabolism has been linked to mitochondrial ROS homeostasis in somatic tissues (Pang & Curran, 2014; Pang et al., 2014; Zarse et al., 2012), but not in the context of spermatogenesis. Without ALH‐6 activity to catabolize P5C, the continuous generation of P5C by PRDH‐1 should lead to redox imbalance and impairment of normal function of germ cells as it does for somatic tissues (Deuschle et al., 2004; Miller et al., 2009; Nomura & Takagi, 2004; Pang & Curran, 2014; Yoon et al., 2004). We hypothesized that if the sperm defects in the *alh*‐*6* mutants are a result of loss of ROS homeostasis, then antioxidant supplementation could alleviate these phenotypes. We supplemented the diet of *alh*‐*6* mutant males with the antioxidant N‐acetylcysteine (NAC), from birth through reproductive maturity, and measured their reproductive parameters. NAC supplementation restored spermatid size (Figure 3a) and activation (Figure 3b) of *alh*‐*6* animals to WT levels, while NAC supplementation in wild‐type (Figure 3c,d) or *alh*‐*6*;*prdh*‐*1* double mutants (Figure 3e,f) had no effect. Similar results were obtained using Vitamin C as an antioxidant (Figures S2a–d). To confirm the role of aberrant ROS homeostasis in driving sperm defects, we exposed male worms to superoxide‐generating compound paraquat and saw that paraquat‐treated worms show an increase in SKN‐1 activity (Figure S2e–f). WT males exposed to paraquat generate spermatids that are smaller (Figure 3g) and display defects in spermatid activation (Figure 3h). In addition, using RNAi on genes involved in endogenous antioxidant defenses in WT males similarly impairs spermatid growth and activation (Figure 3i,j). Interestingly, while multiple antioxidant genes are involved in the reduction in sperm size caused by *alh*‐*6* mutation, only *sod*‐*2* RNAi further exacerbated activation in *alh*‐*6* mutant males (Figure S2 g,h). Altogether, these data reveal that disruption in ALH‐6 activity drives redox imbalance, which leads to defects in sperm health and function.

**FIGURE 4:**
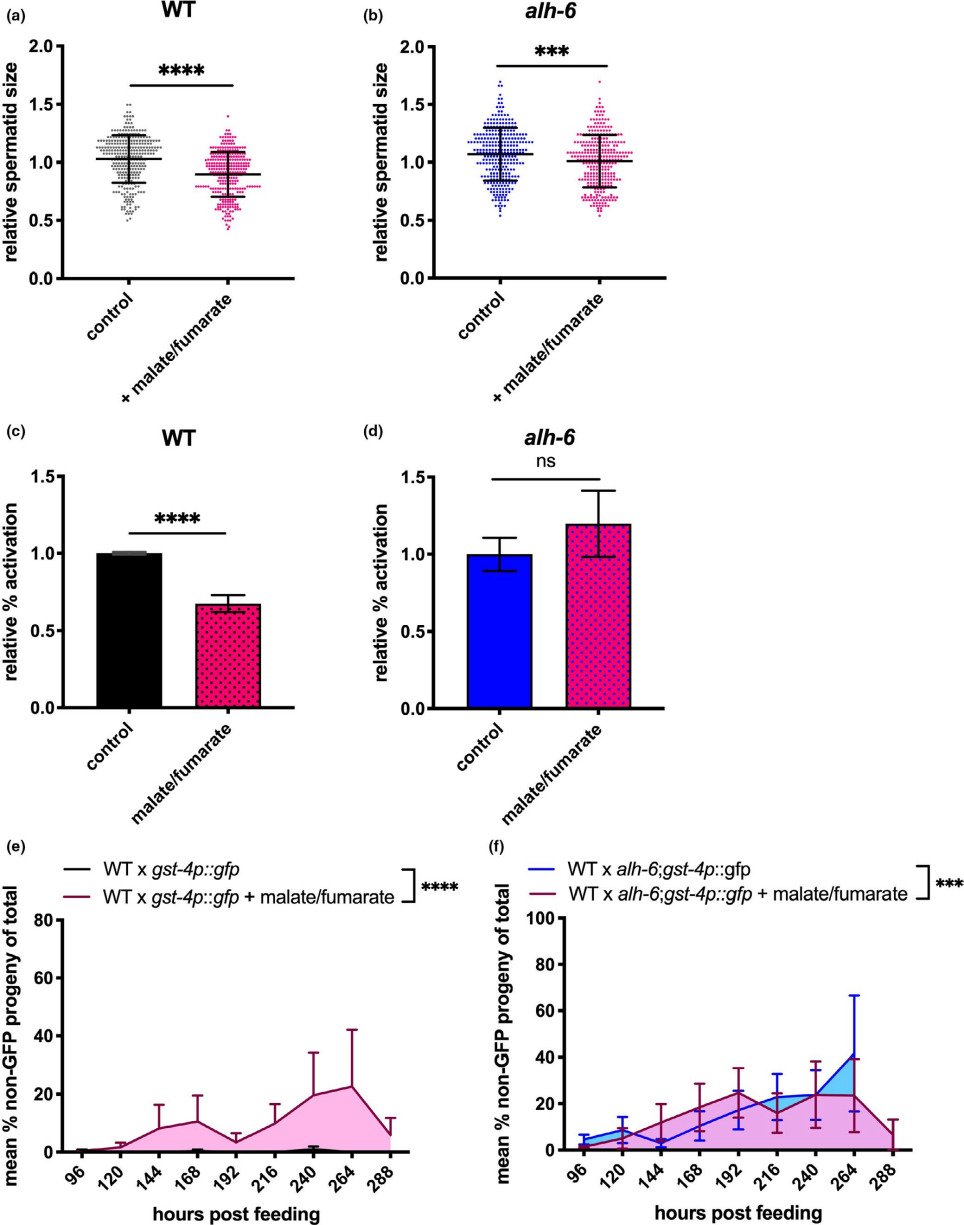
Increasing ETC activity through malate/fumarate supplementation alter sperm function. (a, b) Malate/fumarate supplementation reduces sperm size in WT males (b) and further decreases sperm size of *alh*‐*6* males. (c, d) Malate/fumarate supplement impairs activation in WT male sperm (c), but does not change *alh*‐*6* male sperm (d). (e, f) Malate/fumarate supplement reduces ability for WT spermatids to compete (e), while exacerbating *alh*‐*6* spermatids in competitive ability (f). Comparisons of sperm size were analyzed using unpaired *t*‐tests. Comparisons of sperm activation and competition between unsupplemented diet and diet supplemented with malate/fumarate were done using Fisher's exact test. *, *p* < 0.05; **, *p* < 0.01; ***, *p* < 0.001; ****, *p* < 0.0001. All studies performed in minimum of three biological replicates; refer to Table S1 for n for each comparison

To further examine the role of endogenous ROS on gamete dysfunction, we pharmacologically altered mitochondrial ETC activity. While ETC is essential for cellular respiration, it can also be a major source of metabolism‐generated ROS (Balaban et al., 2005). Increased availability of ETC substrates can alter the rates of oxidative phosphorylation and electron flow through the ETC (Zorov et al., 2014). Specifically, dietary supplementation of malate and fumarate has been demonstrated to decrease electron flow through complex III by activation of NADH‐fumarate reductase (malate dismutation) and passing electrons to rhodoquinone instead of coenzyme Q; which could increase ROS production in vivo when ETC complex inhibitors are absent (Brand, 2010; Edwards et al., 2013). We verified the effects of malate/fumarate supplement on inducing ROS through two methods. Using the *gst*‐*4p*::*gfp* reporter strain, which indicates SKN‐1 activity, we noted an increase in GFP signal in worms that are treated versus those that are not (Figure S2e,f). This increase in SKN‐1 activity in those that are treated versus those that are not is more evident in WT males (Figure S2e), although still visible in the head and tail region of *alh*‐*6* mutant males (Figure S2f). Furthermore, malate/fumarate treatment increased mitochondrial superoxide dismutase *sod*‐*2* and decreased glutathione peroxidase *gpx*‐*5* expression in WT animals compared to those that are not treated (Figure S2i). Interestingly, *alh*‐*6* animals treated with malate/fumarate show an increase in the expression of cytoplasmic and peroxisomal catalases, *ctl*‐*1* and *ctl*‐*2* (Figure S2j). Altogether these data support the observation that malate/fumarate alters metabolism in ways that affect ROS homeostasis. Knowing that malate/fumarate alters expressions of antioxidant enzymes and increases SKN‐1 activity, we next asked if malate/fumarate could affect sperm function. Supplementation of malate/fumarate reduced WT spermatid size, and further decreased *alh*‐*6* mutant sperm size (Figure 4a,b). The addition of this supplement also impairs activation in WT spermatids, while not affecting the already reduced activation rate in *alh*‐*6* male spermatids (Figure 4c,d). Consequently, malate/fumarate supplementation also decreased the ability of both WT and *alh*‐*6* mutant male sperm to compete with hermaphrodite sperm, as shown by the increased usage of self‐sperm in hermaphrodites mated with supplemented males (Figure 4e,f.) Collectively, these data show that supplementation of ETC metabolites can alter antioxidant gene expressions and lead to premature sperm dysfunction; similar to that observed in *alh*‐*6* animals.

**FIGURE 5:**
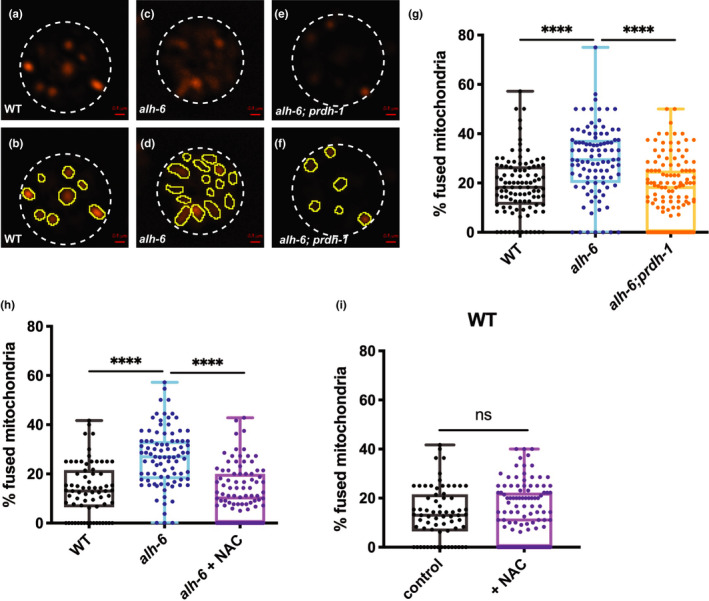
Endogenous ROS drives changes in mitochondria dynamics leading to sperm defects. (a–f) Representative images of JC‐1 dye‐stained mitochondria of WT (a, b), *alh*‐*6* mutant (c, d), *alh*‐*6*;*prdh*‐*1* mutant (e, f) spermatids from dissected males. White dashed lines marked the outside of spermatids. Yellow outlines the individual mitochondria in the spermatids. (g) Quantification of fused mitochondria in images (a–f) shows that *alh*‐*6* mutant male spermatids have increased number of fused mitochondria, which is restored to WT levels in *alh*‐*6*;*prdh*‐*1* mutants. (h) Antioxidant treatment with NAC restores mitochondrial dynamics to wild‐type levels in *alh*‐*6* mutant spermatids. (i) Treatment with NAC does not change mitochondria dynamics of WT spermatids. Comparisons of % fused mitochondria between strains were analyzed using ANOVA, while comparisons for *alh*‐*6* mutant fed control diet to those that are fed on NAC supplemented diet were done using unpaired *t* test *, *p* < 0.05; **, *p* < 0.01; ***, *p* < 0.001; ****, *p* < 0.0001. All studies performed in minimum of three biological replicates; refer to Table S1 for n for each comparison

Recently, we reported that *alh*‐*6* mutant spermatids have increased mitochondrial fusion (Yen et al., 2020). Mutation in *prdh*‐*1*, which restores sperm function (Figure 2), returned spermatid mitochondria to a more punctate and less connected structure that resembles mitochondria in WT spermatids (Figure 5a‐g). Similarly, treatment with the antioxidant NAC returned *alh*‐*6* mutant mitochondrial fusion level in spermatids to WT levels (Figure 5h). Furthermore, NAC treatment does not change the mitochondrial fusion level in WT male spermatids (Figure 5i). Taken together, these data reveal that antioxidant supplementation can act as a treatment to overcome reproductive deficiencies stemming from impaired cellular metabolism.

### 2.5 *alh*‐*6* mutation accelerates male reproductive aging

**FIGURE 6:**
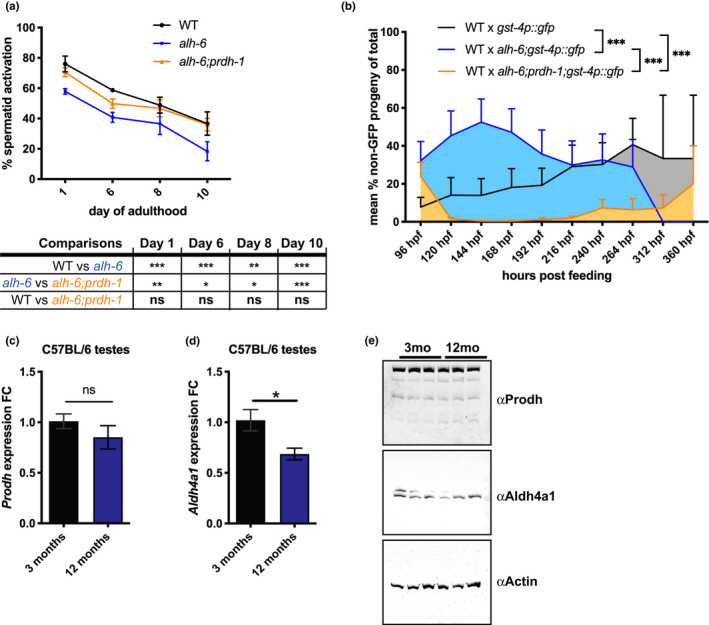
*alh*‐*6* mutation accelerates male reproductive senescence. (a) The ability for sperm to activate upon Pronase treatment declines with age. *alh*‐*6* mutant sperm shows premature decline compared with WT and *alh*‐*6*;*prdh*‐*1*. Statistical comparisons between strains at each age show a significant decrease in ability to activate of *alh*‐*6* mutant spermatid compared with WT, which is restored in *alh*‐*6*;*prdh*‐*1*. (b) The ability for male sperm to compete against hermaphrodite self‐sperm also declines with age, with *alh‐6* mutants showing premature decline compared to WT and *alh‐6;prdh‐1* mutant males. (c, d) RT‐PCR shows unchanged expression of *Prodh* between young and old mice testes (c), while *Aldh4a1* expression is decreased in old mice testes compared with young mice (d). (e) PRODH protein expression is consistent between young and old mice testes, but ALDH4A1 protein expression is reduced in testes of aged mice. Statistical comparisons of sperm activation and competition assay done using Fisher's exact test with p‐value adjusted for multiple comparisons between the groups. *, *p* < 0.05; **, *p* < 0.01; ***, *p* < 0.001; ****, *p* < 0.0001. All studies performed in minimum of three biological replicates; refer to Table S1 for *n* for each comparison

Like most tissues, the reproductive system functionally declines with increasing age. Clinically, age‐related decline in male fertility is diagnosed by a reduction in sperm quality and quantity (Harris et al., 2011). Sperm from older men are reduced in number and motility, and display increased abnormalities, which collectively diminish success in both in vivo and in vitro fertilization (IVF) (Kidd et al., 2001; Mazur & Lipshultz, 2018). In *C. elegans*, the decline in male fertility has been shown in aged males where sperm number is reduced and activation in vitro via Monensin is reduced (Chou et al., 2019). In our hands, the ability for sperm to activate with Pronase treatment (Nelson & Ward, 1980; Yen et al., 2020) also declines with age in wild‐type males (Figure 6a). *alh*‐*6* mutant sperm from day 1 adult males are functionally more similar to WT sperm from day 6 adults, as such, *alh*‐*6* males experience premature reproductive senescence in the form of impaired sperm activation capacity. Importantly, the mutation in *prdh*‐*1* restores the age‐related changes in spermatid activation to wild‐type levels in *alh*‐*6* mutant male sperm, supporting our hypothesis that the accumulation of P5C, in animals with impaired P5C dehydrogenase activity, is causal for age‐related sperm dysfunction.

In order to determine if the accelerated loss of activation was causal for the fertilization defects observed in *alh*‐*6* mutant animals, we performed sperm competition assays as before, but with aged males mated to young hermaphrodites. As expected, WT hermaphrodites mated with day 3 adult WT males resulted in the generation of a small fraction of non‐GFP progeny (indicating usage of hermaphrodite sperm) (Figure 6b) that are not present in day 1 adults (Figure 2f). In contrast, sperm from *alh*‐*6 *day 3 adults was significantly impaired for competition against hermaphrodite self‐sperm resulting in higher incidence of non‐GFP progeny. This deficit in competitive advantage is restored by mutation in *prdh*‐*1* (Figure 6b).

Since *alh*‐*6* mutant worms exhibit age‐related decline in sperm quality, we sought to determine if *Aldh4a1*, the mammalian ortholog of *alh*‐*6*, was regulated by age in the male reproductive system in mice. It is established that oxidative stress increases with age in male testes due to the reduction in both expression and production of antioxidant enzymes in mice (Cao et al., 2004; Luo et al., 2006). We examined the expression of proline catabolism pathway genes *Prodh* and *Aldh4a1* in young (3‐month‐old) and middle aged (12‐month‐old) mice testes. The expression of *Prodh*, was similar (Figure 6c) between 12‐month‐old and 3‐month‐old mice testes, but the expression of *Aldh4a1* was significantly reduced in the older mice testes (Figure 6d). Furthermore, expression of both *Prodh* and *Aldh4a1* remains similar in the livers of 3‐month‐old and 12‐month‐old mice, showing that the differential expression of *Aldh4a1*, but not *Prodh*, in older mice testes is tissue‐specific (Figure S3a,b). In support of this finding, the steady‐state protein level of PRODH is similar in both testes and livers of 3‐month‐old and 12‐month‐old mice, while total ALDH4A1 enzyme levels are reduced in older testes, but unchanged in young and older livers (Figure 6e, Figure S3c). Reduction of ALDH4A1 without a corresponding decrease in PRODH could lead to the accumulation of P5C, a scenario that could lead to sperm dysfunction as we found in worms (Yen et al., 2020).

Lastly, *alh*‐*6* mutation in worms drives premature health decline in the soma that ultimately leads to a shortened lifespan, although these effects are not observed until the end of the reproductive period (~day 5 of adulthood) (Pang & Curran, 2014). Since mutation in *alh*‐*6* results in premature aging, we wondered if *prdh*‐*1* mutation could mitigate the shortened lifespan phenotype of *alh*‐*6* hermaphrodites (Figure S4a). Although *prdh*‐*1* mutation suppressed the activation of the SKN‐1 reporter and the sperm defects of *alh*‐*6* mutants, we found that *alh*‐*6*;*prdh*‐*1* double mutants do not live significantly longer than *alh*‐*6* mutants (Figure S4b). Our attempts to measure the impact of mutations in *alh*‐*6* and *prdh*‐*1* on male lifespan was confounded by the hyper‐exploratory behavior of males (Barrios et al., 2008; Kleemann et al., 2008), which drives high censor rates (Figure S4c). However, we did note a significant reduction in exploration‐dependent censoring of *alh*‐*6* males that was reversed in the *alh*‐*6*;*prdh*‐*1* double mutant (Figure S4c). Notably, WT and *alh*‐*6*;*prdh*‐*1* males continue to crawl up the sides of the wells up to day 20 of adulthood, while *alh*‐*6* males do not engage in as much exploratory behavior (Figure S4d). These data reveal a physiological outcome tied to the established loss of muscle homeostasis previously reported for *alh*‐*6* mutants (Pang & Curran, 2014). Nevertheless, these results suggest that *prdh*‐*1* may play roles in somatic functions that contribute to the health of the animal that are distinct from the role *prdh*‐*1* and *alh*‐*6* play in spermatogenesis described in this study.

Although further examination is required to understand the long‐term physiological effects of *prdh*‐*1* mutation, taken together, these data reveal a novel role for *alh*‐*6* in the regulation of male reproductive senescence that is dependent on the first committed step of proline catabolism through *prdh*‐*1*. Mutation in *alh*‐*6* results in metabolic changes aimed to restore homeostasis, the result of which leads to redox imbalance and accelerated aging in the germline. Importantly, these sperm defects can be modulated by altering the expression of the proline catabolism pathway genes or by treatment with dietary antioxidants. Finally, the loss of competitive advantage in germ cells of male with incomplete proline catabolism has implications for overall fitness of the organism.

## 3 DISCUSSION

Although nearly half of idiopathic infertility cases are thought to have a genetic basis with lifestyle as contributing factors (Zorrilla & Yatsenko, 2013), their underlying mechanistic basis is largely unknown. While the importance of maternal age in female reproductive capacity has been highlighted in many studies (Klein & Sauer, 2001), the role of paternal age in fertility has received less attention. Our previous study identified FAD homeostasis and changes in mitochondrial metabolism and dynamics as mechanisms that regulate male fertility in *C. elegans* (Yen et al., 2020). This study expands our understanding of how altering endogenous metabolic processes can affect sperm aging.

It is perhaps surprising that the complete reduction in mitochondrial proline catabolism (mutation in both *prdh*‐*1* and *alh*‐*6*) is relatively benign. We discovered that a mutation in the upstream proline dehydrogenase pathway gene, *prdh*‐*1*, is able to suppress the sperm defects observed in *alh*‐*6* mutants (Figure 2), revealing that the overall reduction in proline catabolism is not causal for the observed reproductive phenotypes, but rather, the reduced ability to convert P5C, which would accumulate in *alh*‐*6* mutants, to other more stable intermediates. Interestingly, our analysis of gene expression changes in proline metabolism genes revealed a downregulation of *alh*‐*6* (loss of P5C dehydrogenase), but an upregulation of *pycr*‐*1* and *alh*‐*13* (increase in proline biosynthesis) in *alh*‐*6* mutants at L4 stage (Figure 1h). In other species, metabolic adaptation to environmental stresses has been shown to occur through transcriptional regulation of proline biosynthetic and catabolic genes (Liang et al., 2013). In fact, proline has been shown to accumulate in response to oxidative stress through upregulation of its biosynthetic pathway, and cells deficient in P5C reductase are more sensitive to ROS (Phang, 2019). Moreover, since proline itself has important roles in cellular protection, the increased expression of its biosynthetic genes may be an important stress response, but with pleiotropic consequences as it would deplete glutamate and increase an already accumulating pool of P5C.

Our studies reveal that the transcription of proline catabolism pathway genes is under temporal regulation, such as *pycr*‐*1* and *alh*‐*13*, which are expressed almost exclusively during development (Figures S1e–j). *C. elegans* express a second isoform of PYCR, *pycr*‐*4*, which is expressed in distinct tissues, is more uniformly expressed at both L4 stage and day 3 of adulthood, and is less responsive to impaired *alh*‐*6* and *prdh*‐*1* (Wormbase, 2006). Intriguingly, the expression of PRDH‐1 and ALH‐6 are reduced, in concert, with age while loss of *alh*‐*6* function results in a reduction of only *alh*‐*6* and no change in *prdh*‐*1* expression; a scenario that would drive accumulation of P5C. These findings suggest that the mitochondrial proline catabolism pathway genes are under transcriptional regulation, but further studies to identify their transcriptional mediators are needed. Moreover, future studies to measure enzymatic activities of proline metabolism pathway enzymes during normal aging and in the context of mutant backgrounds will be useful.

In humans, hyperprolinemia (HP) types I (*PRODH)* and II (*ALDH4A1)* are both diagnosed by elevated level of proline in plasma, with addition of high level of P5C in HPII patients. This increase in proline level in HPII may be caused by transcriptional changes in the proline metabolism pathway similar to what we see in our *alh*‐*6* mutant worms (Figure 1h,i).The symptoms of HPI varies in severity depending on the degree of reduction in PRODH activity, and are characterized by neurological, auditory, and renal defects, while symptoms of HPII are variable and characterized by neurological defects (Geraghty et al., 1998). Due to the limited number of clinically diagnosed HPI and HPII patients, there are relatively few studies on the molecular basis of hyperprolinemia. Furthermore, there is a lack of understanding in the long‐term effects of this syndrome, as well as any current effective treatment for patients (Mitsubuchi et al., 2014). Interestingly, in a recent study of HPII patients, mitochondrial dysfunction in fibroblasts from muscle biopsy was suggested, although limited by small sample size (one out of five patients) (van de Ven et al., 2014). This muscle mitochondrial phenotype in humans is reminiscent of the fragmented muscle mitochondria we previously characterized in *alh*‐*6 *day 5 adult worms (Pang & Curran, 2014).

Although proline catabolism has not been shown to have a direct role in fertility, studies in primate and other species have shown that the addition of proline in cryopreservation medium improves sperm mobility and preservation of membrane integrity upon thawing (Li et al., 2003). Notably, the brood size between WT and *alh*‐*6*;*prdh*‐*1* double mutant is not significantly different, although *alh*‐*6*;*prdh*‐*1* double mutant animals routinely yield fewer progeny (Figure 2c). Furthermore, the brood size between *alh*‐*6*;*prdh*‐*1* and *alh*‐*6* mutant is also not statistically different, although there is a trend toward an increase in number of progeny in the double mutant. Recent studies in mice have shown that the addition of proline increases the viability of vitrified oocytes by acting as a cryoprotectant (Zhang et al., 2016). As such, proline metabolism may also affect oocyte health in *C. elegans* hermaphrodites in addition to the effects on sperm reported here.

About 30–40% of all male infertility cases are associated with increased levels of ROS (Jose‐Miller et al., 2007). Additionally, ROS generation increases as sperm quality decreases with age (Cocuzza et al., 2008; Kidd et al., 2001). Our study demonstrates that the impaired sperm function stemming from perturbation of mitochondrial proline catabolism, specifically mutation in *alh*‐*6*/*ALDH4A1*, leads to increased ROS and can be alleviated pharmacologically by antioxidant treatments (NAC and Vitamin C). Using RNAi to limit the expression of antioxidant genes, we demonstrate that loss of ROS homeostasis can also affect sperm quality. Our study reveals that both *sod*‐*1* and *sod*‐*2* affect the rate of activation in WT sperm, while only *sod*‐*2* RNAi further exacerbates the defective activation in *alh*‐*6* mutant sperm. Interestingly, *sod*‐*2 *has been recently shown to be required to produce H_2_O_2_ to induce sperm activation via Pronase (Sakamoto & Imai, 2017), which demonstrates the need to tightly regulate ROS homeostasis for germ cell development. Future investigation to directly measure how antioxidant treatments impact age‐related loss of reproductive capacity in mammals will be of great interest.

Lastly, we found that expression of *Aldh4a1* in mice testes also declines with age, while *Prodh* remains unchanged (Figure 6c,d). Using western blot analysis, we found that a slower migrating species of ALDH4A1 is decreased in older mice testes, while PRODH level remains unchanged between young and older mice testes (Figure 6e).These expression changes in proline metabolism pathway proteins in old mice testes mirrors the genetic perturbation of *alh*‐*6* in our worm model. Although there is no prior evidence of *Aldh4a1* in regulating male fertility in mice, *Aldh4a1 *has been shown to be significantly downregulated in oocytes isolated from post‐reproductive mice compared with young female mice (Esteves et al., 2011). It may be interesting to investigate whether proline metabolism plays a direct role in mammalian sperm health and whether mechanisms identified in our studies are conserved. If conserved, *Aldh4a1* may act as a potential genetic biomarker in the diagnosis and treatment of male infertility. Antioxidant supplements could be studied as part of comprehensive treatment plans in cases identified by genetics, which are currently not common practice but may gain traction as sequencing costs decrease and the genetic basis of male infertility is better defined. Taken together, our studies define an important and conserved role for mitochondrial proline catabolism in male reproductive fitness, which establishes a new target and approach for combating male infertility.

## 4 EXPERIMENTAL PROCEDURES

### 4.1 *Caenorhabditis elegans* strains and maintenance

*Caenorhabditis elegans* were cultured using standard techniques at 20°C. The following strains were used: wild‐type (WT) N2 Bristol, CB4856 (HW), SPC402[*alh*‐*6(lax105)*;*gst*‐*4p*::*gfp*;HW], SPC494[*alh*‐*6(lax105)*;*prdh*‐*1(lax228)*], SPC321[*alh*‐*6(lax105)*], CL2166[*gst4*‐*p*::*gfp*], SPC223[*alh*‐*6(lax105)*;*gst*‐*4p*::*gfp*], SPC490[*alh*‐*6(lax105)*;*prdh*‐*1(lax228)*;*gst*‐*4p*::*gfp*], and SPC493[*alh*‐*6(lax105)*;*prdh*‐*1(lax228)*;*gst*‐*4p*::*gfp laxEx7*(*prdh*‐*1p*::*prdh*‐*1*;;*myo*‐*2p*::*rfp*;*rab*‐*3p*::*rfp)*]. Double and triple mutants were generated by standard genetic techniques. *E*. *coli* strains used were as follows: B Strain OP50 (Brenner, 1974) and HT115(DE3) [F^−^mcrA mcrB IN(rrnD‐rrnE)1 lambda^−^ rnc14::Tn10 γ(DE3)] (Timmons et al., 2001). For dietary supplement assays, the following was added to the NGM plate mix to final concentration: 5 mM NAC,10 mM Vitamin C, 5 mM malate, 5 mM fumarate, and 75 uM paraquat.

### 4.2 EMS mutagenesis

Ethyl methanesulfonate mutagenesis was performed as previously described (Paek et al., 2012). Briefly, SPC223[*alh*‐*6(lax105)*;*gst*‐*4p*::*gfp*] was mutagenized with EMS and F2 worms with reduced GFP expression (indicating suppression of SKN‐1 activation) were selected. *prdh*‐*1(lax228)* was isolated and mapped to chromosome IV. Whole‐genome sequencing and injection rescue confirmed mutant sequence identity.

### 4.3 Whole‐genome sequencing

Worms were treated with alkaline hypochlorite and eggs were allowed to hatch overnight. The next day 3000–4000 synchronized L1s were dropped on NGM plates seeded with 25X concentrated OP50. After 48 h, L4 animals were washed three times with M9, homogenized, and genomic DNA was extracted using Zymo Quick‐DNA Miniprep Kit. DNA samples were library prepped and sequenced by USC Epigenome Center Data Production Facility. Sequencing data were analyzed using Galaxy.

### 4.4 Lifespan analysis

Lifespan assays were performed as previously described (Pang & Curran, 2014). Worms were treated with alkaline hypochlorite and eggs were allowed to hatch overnight. The next day, synchronized L1 larvae were dropped on NGM plates seeded with OP50. 48 h later, 50 L4 hermaphrodites were moved individually onto new plates in replicates of three for each genotype. Worms were transferred every day during the reproductive period. Worms that died of vulva burst, bagging, or crawling off the plates were censored. For male lifespan analyses, males were singled as L4s onto each well of 12‐well plates (total of four plates for each replicate) and moved every 3 days to fresh plates. Plates were scored every day for dead animals. Worms that crawled off the plates or could not be found for two consecutive days were censored.

### 4.5 Fertility assay

Worms were treated with alkaline hypochlorite, and eggs were allowed to hatch overnight. The next day, synchronized L1 larvae were dropped on NGM plates seeded with OP50. 48 h later, at least 10 L4 hermaphrodites for each genotype were singled onto individual plates and moved every 12 h until egg laying ceased. Unfertilized oocytes were counted 24 h after the singled hermaphrodite was moved and progeny were scored 48 h after the singled hermaphrodite was moved to a different plate. Plates were counted twice for accuracy.

### 4.6 Mated reproductive assay

Males were synchronized by egg laying, picked as L4 larvae for use as young adults for mating experiments. Singled L4 stage hermaphrodites were each put on a plate with 30 µl of OP50 seeded in the center together with three virgin adult males. 24 h post‐mating, males were removed, and each hermaphrodite was moved to a new plate every 24 h until egg laying ceased. Progeny were counted 48 h after the hermaphrodite was moved from the plate. For sperm competition assay, progeny with GFP fluorescence were counted and removed from plates before non‐GFP progeny were counted. For aged male GFP mated reproductive assay, age‐synchronized day 3 adult virgin males were used to mate to L4 WT hermaphrodites and the protocol above was followed post‐mating. All plates were counted twice for accuracy.

### 4.7 Sperm number assay

Worms were treated with alkaline hypochlorite and eggs were allowed to hatch overnight. The next day, synchronized L1s were dropped on NGM plates seeded with OP50. 72 h post‐drop, day 1 adult hermaphrodite animals were washed 3x with 1xPBST, fixed with 40% 2‐propanol, and stained with DAPI for 2 h. Samples were washed for 30 min with PBST, mounted with Vectashield mounting medium, and covered with coverslip to image. Spermatids in spermathecae of both gonad arms were counted through all planes in z‐stack.

### 4.8 Sperm size assay

Males were isolated at L4 stage 24 h before assay. For each strain, five day 1 adult males were dissected in 35 µl pH 7.8 SM buffer (50 mM HEPES, 50 mM NaCl, 25 mM KCl, 5 mM CaCl_2_, 1 mM MgSO_4_, 10 mM dextrose) to release spermatids, which were immediately imaged.

### 4.9 Sperm activation with pronase

Males were isolated at L4 stage 24 h before assay. For each strain, five day 1 adult males were dissected in 35 µl pH 7.8 SM buffer (50 mM HEPES, 50 mM NaCl, 25 mM KCl, 5 mM CaCl_2_, 1 mM MgSO_4_, 1 mg/ml BSA) supplemented with 200 µg/ml Pronase^®^ (Millipore Sigma) to release spermatids. Another 25 µl of the same solution was added and the spermatids were incubated at RT for 8 min for activation to occur before imaging. Sperm medium was made fresh for each experiment and optimal incubation time determined empirically by observing the time required for WT spermatids to reach 80–90% activation. For aged male sperm activation assays, individual L4 virgin males were singled onto plates for each strain and moved every 3 days onto fresh plates. Due to high censor rate while aging males, a higher number of males are required to start with when singling at L4 stage.

### 4.10 Sperm mitochondria staining

Males were isolated at L4 stage 24 h before assay. For each strain, five day 1 adult males were dissected in 35 µl pH 7.8 SM buffer (50 mM HEPES, 50 mM NaCl, 25 mM KCl, 5 mM CaCl_2_, 1 mM MgSO_4_, 1 mg/ml BSA) with JC‐1(Thermo Fisher Scientific T3168) added to 15 µM final concentration. Another 25 µl of the same solution was added and the spermatids were incubated at RT for 10 min. The slide was washed three times with 100 µl SM buffer before imaging.

### 4.11 RT‐PCR

Worms were treated with alkaline hypochlorite and eggs were allowed to hatch overnight in M9. The next day, 3000–4000 synchronized L1s were dropped on NGM plates seeded with 25X concentrated OP50. After 48 and 120 h, L4 animals and day 3 adult animals, respectively, were washed three times with M9 and frozen in TRI Reagent at −80°C. Male C57BL/6 mice were dissected at 3‐month‐ and 12‐month‐old (5 each) and their testes and liver tissues that are frozen in TRI Reagent. Worms or mice tissues were homogenized and RNA extraction was performed following the protocol in Zymo Direct‐zol RNA Isolation Kit. RNA samples were used to make cDNA using qScript cDNA Supermix (Quantabio). cDNA is mixed with PerfeCTa SYBR Green FastMix (Quantabio) and primers of target genes to analyze their expression on Biorad CFX96 Touch Real‐Time PCR Detection System. Target gene expressions were normalized to *snb*‐*1* in analysis. Statistical comparisons of two groups were analyzed using unpaired *t*‐test, while ANOVA was used for comparing groups of three or more.

### 4.12 Western blot

Three‐month‐old and 12‐month‐old mice tissues were flash frozen in liquid nitrogen and stored at −80°C before use. Tissues were briefly homogenized in T‐PER (Thermo Fisher) and centrifuged at 10,000 × g for 5 min to remove insoluble material. Protein concentration was determined by Bradford assay. Protein extracts were denatured at 95°C for 5 min and separated by gel electrophoresis (Thermo Fisher) and transferred to nitrocellulose membranes. Membranes were blocked in 5% milk in 1X PBST for 1 hour at room temperature. Membranes were incubated overnight at 4°C with primary antibodies specific to PRODH (Abcam ab203875), ALDH4A1 (Abcam ab181256), Histone H3 (Sigma H0164), Actin (Sigma A3853), washed and incubated with goat anti‐rabbit secondary antibody (Abcam) at room temperature for 1 h, and protein visualized with SuperSignal (Thermo Fisher) chemiluminescent substrate.

### 4.13 Microscopy

Zeiss Axio Imager and ZEN software were used to acquire all images used in this study. For GFP reporter strains, worms were mounted in M9 with 10 mM levamisole and imaged with DIC and GFP filters. For sperm number, assay samples were imaged with DIC and DAPI filters in z‐stacks. For sperm size and activation assays, dissected sperm samples were imaged at 100x with DIC filter on two different focal planes for each field to ensure accuracy. For sperm mitochondria assays, dissected sperm samples were imaged at 100x with DIC, GFP, and RFP filters in z‐stacks to assess overall mitochondria content within each spermatid.

### 4.14 Statistical analysis

Data are presented as mean ± SEM. Comparisons and significance were analyzed in GraphPad Prism 8. Comparisons between two groups were done using unpaired *t*‐test. Comparisons between more than two groups were done using ANOVA. For sperm activation assays and mated reproductive assay with GFP reporter males, Fisher's Exact Test was used and *p*‐values were adjusted for multiple comparisons for groups of 3. *p*‐value of less than 0.05 is considered significant (*p < 0.05 **p < 0.01 *** *p* < 0.001 ****p < 0.0001).

## CONFLICT OF INTERESTS

The authors declare that they have no conflicts of interest with the contents of this article.

## AUTHOR CONTRIBUTIONS

S.P.C. designed the study. C‐A.Y. performed the experiments. C‐A.Y. and S.P.C. analyzed data. C‐A.Y. and S.P.C. wrote and revised the manuscript.

## Supporting information

Figure S1

Figure S2

Figure S3

Figure S4

Table S1

Table S2

## Data Availability

All data are contained within the manuscript.
