# Supplementary material for: Incomplete proline catabolism drives premature sperm aging
Source: Aging Cell. 2021 Jan 21;20(2):e13308. doi: 10.1111/acel.13308 (PMC7884046; doi:10.1111/acel.13308)
Supplement: Supplementary file 1 — Figure S1 [file ACEL-20-e13308-s001.pdf]

(a)

Gene ID	Codon change	Protein sequence changed
F56D5.1	gGt/gAt	G149D
R11A8.1	Ggt/Agt	G242S
K08E4.1	Ctt/Ttt	L1174F
C39E9.9	Gaa/Aaa	E246K
Y105C5A.24	Ctt/Ttt	L388F
C06A12.3	aCa/aTa	T22I
B0513.5	Gtg/Atg	V124M

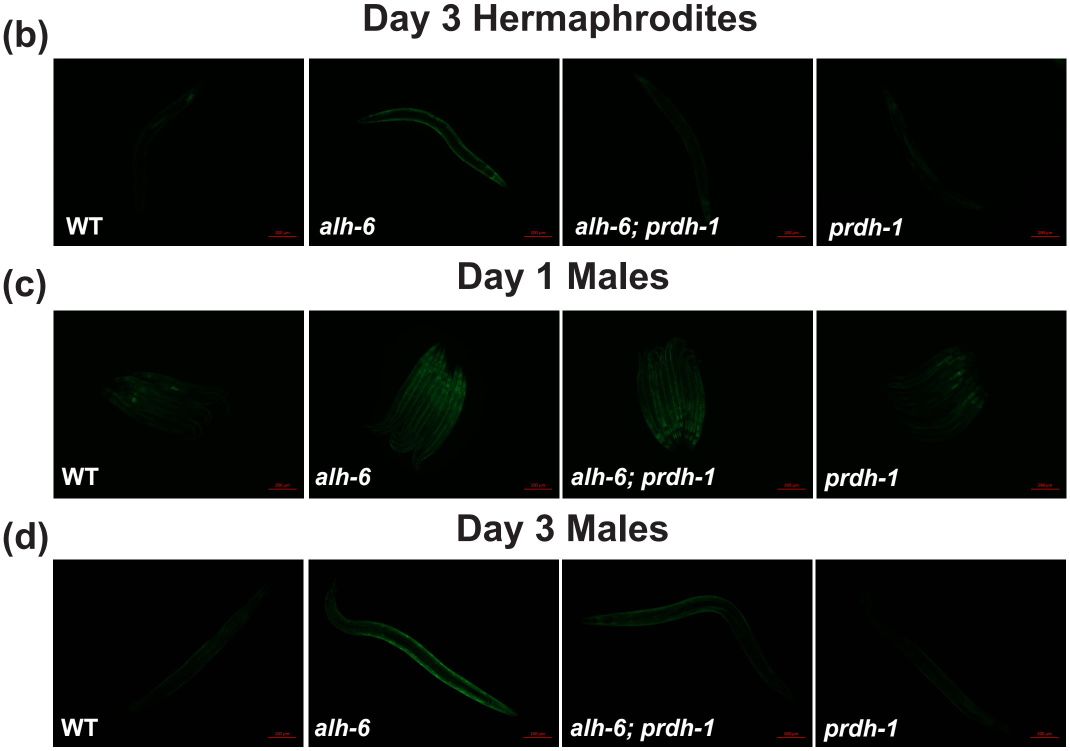

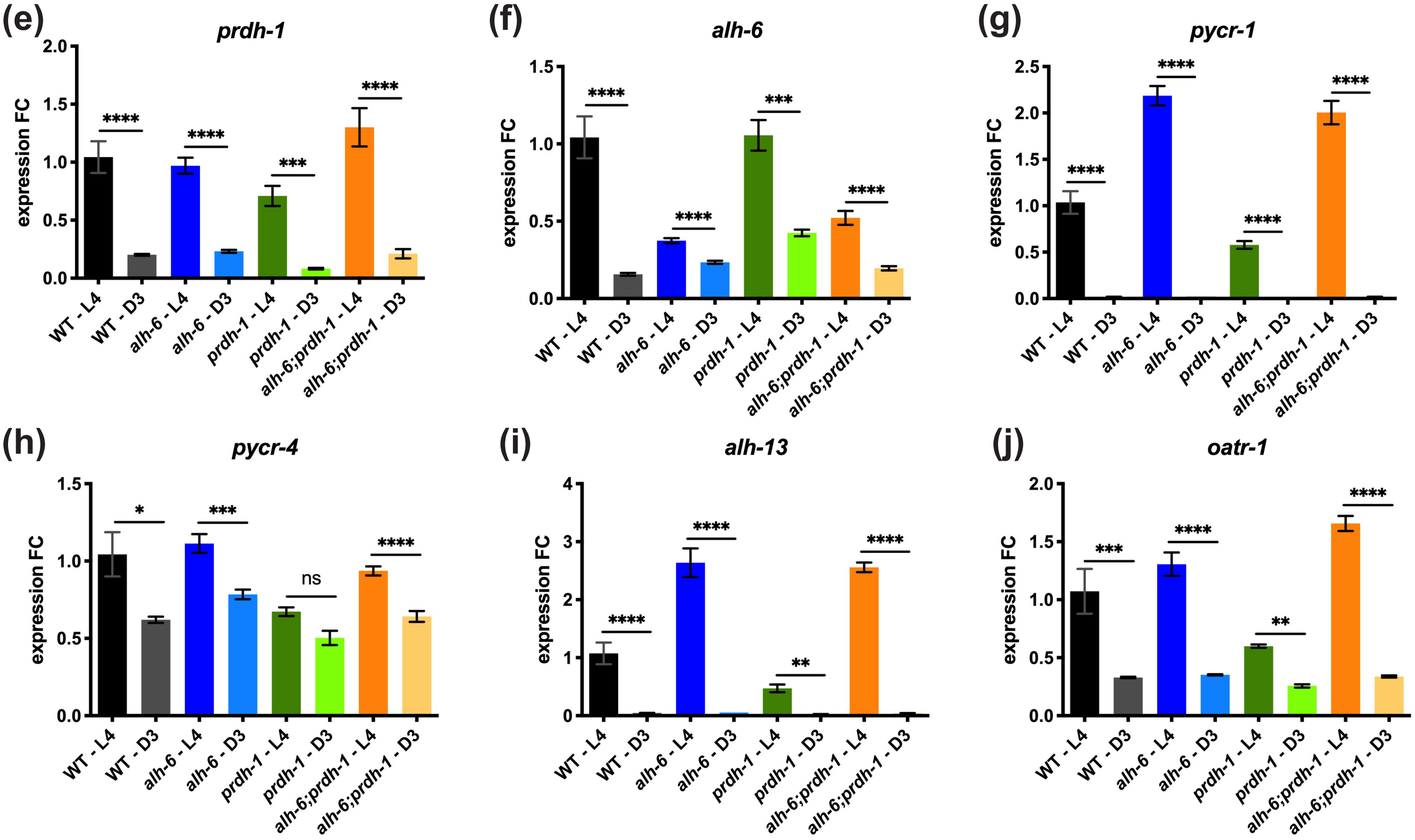

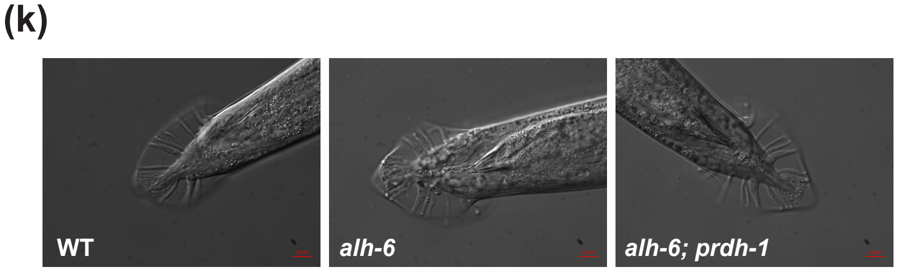

Yen PRDH-1
Supplemental Figure 1
