# Supplementary figures and images for: Incomplete proline catabolism drives premature sperm aging
Source: Aging Cell. 2021 Jan 21;20(2):e13308. doi: 10.1111/acel.13308 (PMC7884046; doi:10.1111/acel.13308)

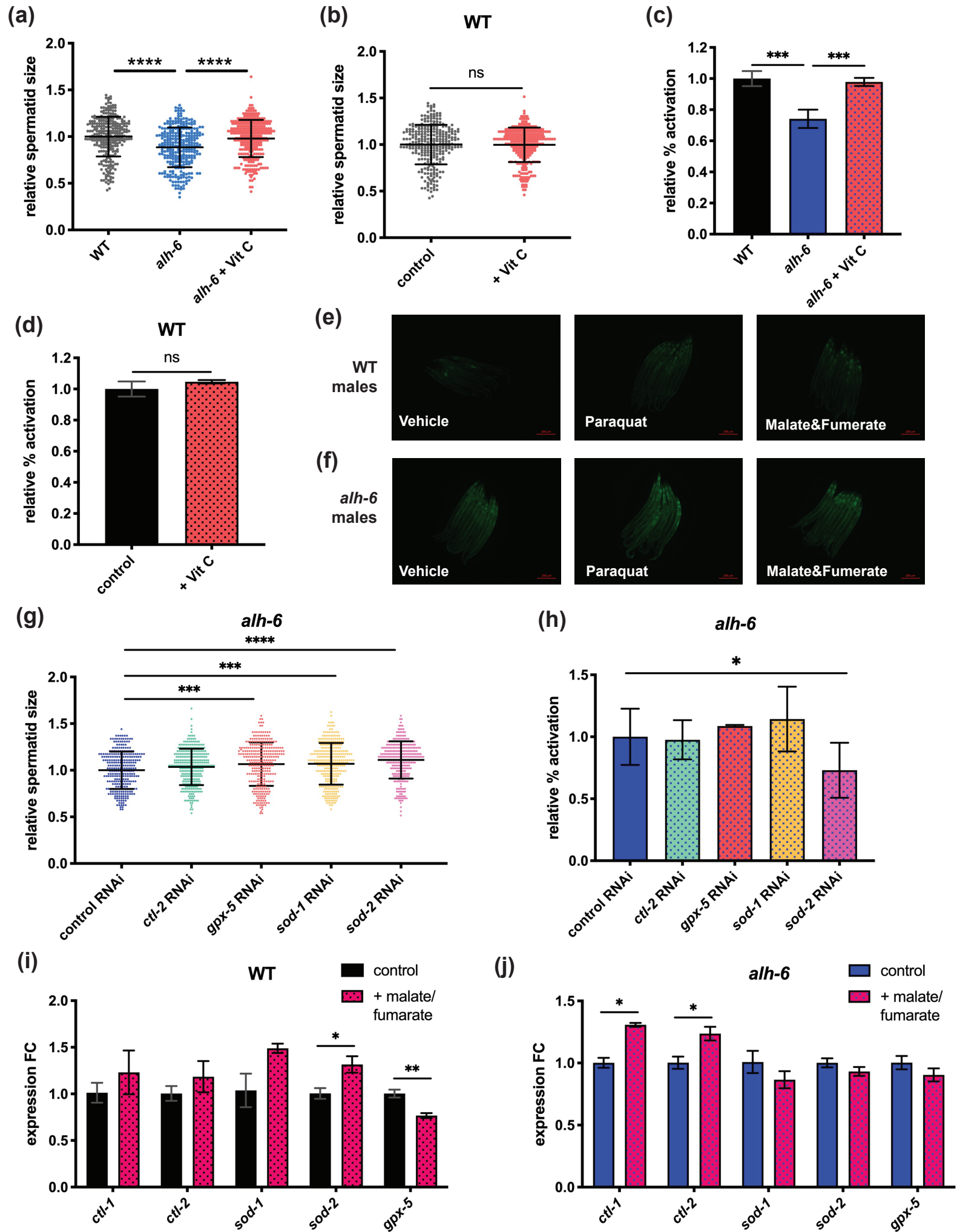

Supplement: Supplementary file 2 — Figure S2 [file ACEL-20-e13308-s002.pdf]

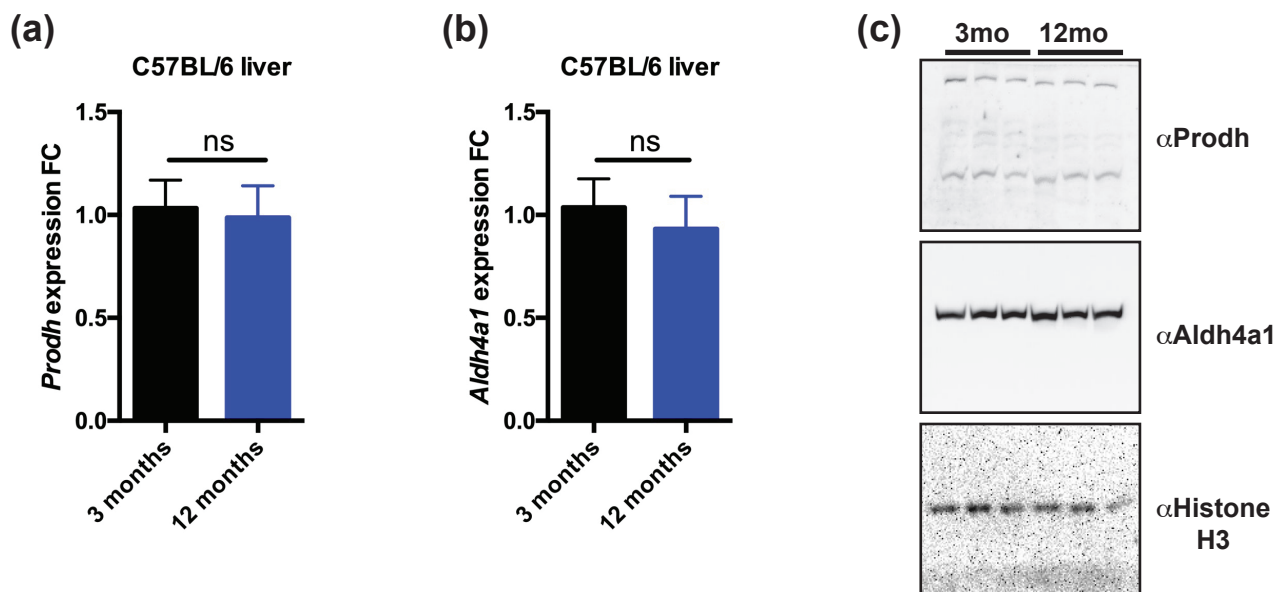

Supplement: Supplementary file 3 — Figure S3 [file ACEL-20-e13308-s003.pdf]

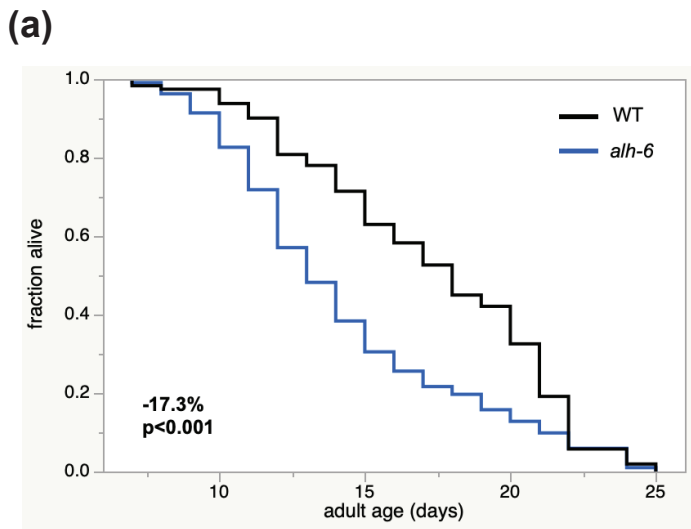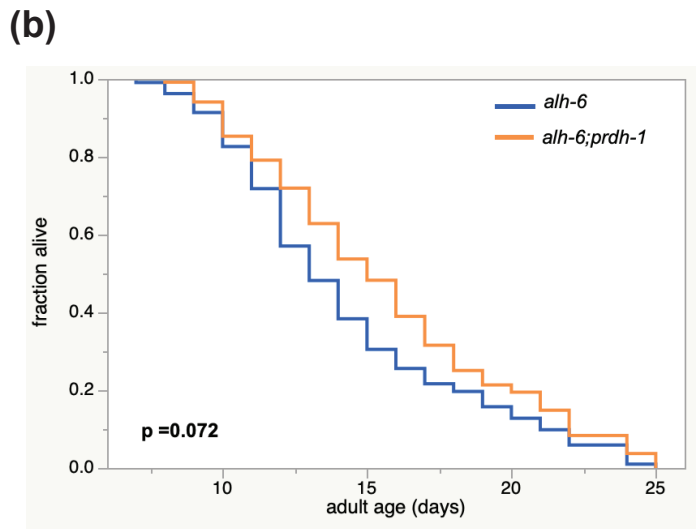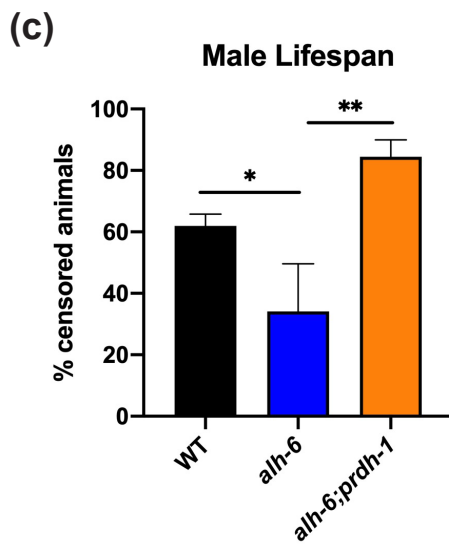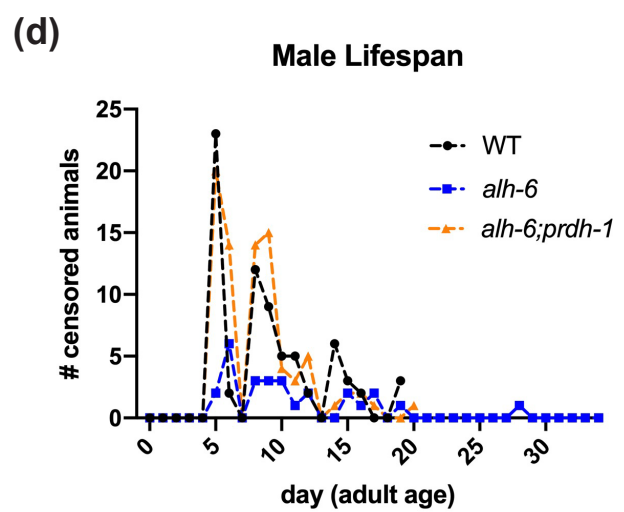

Supplement: Supplementary file 4 — Figure S4 [file ACEL-20-e13308-s004.pdf]
